# Influence of Sociodemographic, Behavioral and Other Health-Related Factors on Healthy Ageing Based on Three Operative Definitions

**DOI:** 10.1007/s12603-019-1243-5

**Published:** 2019-08-30

**Authors:** Agnieszka Pac, B. Tobiasz-Adamczyk, P. Błędowski, A. Skalska, A. Szybalska, T. Zdrojewski, A. Więcek, J. Chudek, J.-P. Michel, T. Grodzicki

**Affiliations:** 1 Chair of Epidemiology and Preventive Medicine, Jagiellonian University Medical College, Krakow, Poland; 2 Institute of Social Economy, Warsaw School of Economics, Warsaw, Poland; 3 Department of Internal Medicine and Gerontology, Jagiellonian University Medical College, Krakow, Poland; 4 International Institute of Molecular and Cell Biology, Warsaw, Poland; 5 Department of Arterial Hypertension and Diabetology, Medical University of Gdansk, Gdańsk, Poland; 6 Department of Nephrology, Transplantation and Internal Medicine, Medical University of Silesia, Katowice, Poland; 7 Department of Pathophysiology, Medical Faculty in Katowice, Medical University of Silesia, Katowice, Poland; 8 Geriatric Department, University of Geneva, Geneva, Switzerland

**Keywords:** Healthy ageing, older people, PolSenior, functioning, lifestyle

## Abstract

**Objectives:**

Healthy ageing (HA) is a key concept and highly desirable phenomenon in every ageing and already old societies. The aim of our study was to evaluate the influence of socio-economic conditions as well as life-style and other health-related factors on the WHO definition of HA.

**Design, Setting, Participants:**

The study used cross-sectional data of the PolSenior Project — nationwide research evaluating different aspects of ageing in Poland — which included 4’653 respondents aged 65 years and over.

**Measurements:**

Data were collected by trained interviewers in respondents’ homes. Three definitions of HA including or not the participants’ chronic conditions were analyzed.

**Results:**

The prevalence of HA appeared as high as 17.6% if none or 1 chronic disease was present and 42.8% if no information about chronic diseases was taken into account. The association between known health predictors (age, marital status, education, income) and HA was observed. Moreover, HA appeared in relation with indicators of physical functioning and lifestyle. There was a strong concordance between HA and the fair self-rated health (OR = 1.87; 1.99, and 2.74 for the 1st, 2nd and 3rd definitions, respectively) and opposite relation with self-reported need for help (OR = 0.15; 0.15; and 0.13, respectively).

**Conclusions:**

The HA definition based on no functional activity limitations, no cognitive impairment, no depressive symptoms, no more than one disease and being socially active seems to be a useful approach of HA..

**Electronic Supplementary Material:**

Supplementary material is available for this article at 10.1007/s12603-019-1243-5 and is accessible for authorized users.

## Introduction

Increasing life expectancy in the most developed societies and growing number of older people significantly influence questions concerning the healthy or successful ageing. The last decades have brought several concepts of healthy or successful or active ageing but still no consensual definition has not been developed (1–3). Generally, all those concepts are understood as ageing well or having a “good” or independent old age and are highly desirable phenomenon both individually and socially (4). Lack of consistency in existing definitions is a fundamental weakness for the state of research in this area (1) and cause several difficulties in assessment the level of healthy ageing in various population of older people; depending on the criteria used different prevalence of healthy/successful ageing has been noticed. Most of existing definitions are based or extend classical definition developed by Rowe and Kahn (5) which stated the successful ageing as a balance of three components: absence of disease and disease-related disability, high functional capacity, and active engagement with life (5, 6). In practice, for the purpose of performing empirical studies, specific operational definitions have been developed based on different models like physiological, engagement or well-being constructs. Most of current perspectives have defined the healthy ageing from the viewpoint of multidimensional approach connecting biomedical aspects with psychosocial dimensions of ageing (3, 7).

Some researchers, as well as older people themselves, suggest that the presence of any chronic disease is not clearly related to healthy ageing, because most of older respondents have some disease and they are “used to” inconvenience related to this disease. The problem might be more visible if the disease strongly influences on everyday activities, or may be source of pain or some psychological disorders (4, 8). World Health Organization (WHO) defines healthy ageing as “the process of developing and maintaining the functional ability that enables wellbeing in older age”, where “functional ability comprises the health-related attributes that enable people to be and to do what they have reason to value” (7). This is also confirmed when older people speak about themselves — the study of Bryant et al. (9) showed that “healthy” means having something worthwhile and desirable to do, obtaining the necessary resources for it, possessing abilities to meet challenges and having will to do it. Healthy ageing depends on genetic, environmental and behavioural factors, as well as socioeconomic determinants. The very important is a point of view of older respondents.

Empirical studies based on different definitions of healthy/successful ageing showed different result depending on population included from 3% to even half of the population studied (7, 10–14). One of the lowest prevalences of successful ageing was found in the US population in “Health and Retirement study” (10) and among Western Mexico elderly population — 12.6% of older people were “successfully” ageing (13). Data coming from Canadian Heath Survey confirmed successful ageing in 11.0% older adults and moderate successful ageing in 77.6% subjects (6); similar results were found in National Survey of the Mental Health and Quality of life of Older Malaysians (14). Very high rate of successful ageing was observed in Shanghai, China 46.2% (95%CI: 43.5%–48.7%) among people aged 65 yrs. or over, but much lower for elders aged 85 yrs. or over (9.4%) (11), and even higher in the healthy community-dwelling Brazilians in age 60 years and older 62% (12). In a longitudinal British civil service study — based on cohort followed for 17 yrs., successful ageing was defined as a being free of major diseases, and in the top tertile of physical and cognitive functioning measured in 2002–2004 and it showed 12.8% men and 14.6% women being successfully ageing (15). Cross-national data coming from SHARE Project (16) showed variation in successful ageing among European countries, and in comparison to US — the highest prevalence was observed in older Danes (21.2%) in relation to a mean value of 8.5%, but the lowest was in Poland (only 1.6%). Opposite to this data — other study based on the SHARE project, using data for 6 countries, including Poland showed that 47.1% of men and 41.0% of women were classified as presenting healthy ageing (17). These data show that prevalence of healthy/successful ageing can be strongly dependent not only on the population but also on the definition used by authors.

The primary objective of our study was to evaluate the influence of socio-demographic and behavioral factors and other health-related indicators as described in the WHO Report (7) on healthy ageing based on different definitions.

## Material and methods

The presented analysis was carried out using data gathered in PolSenior Project, which was the first nationwide cross-sectional research evaluating medical, psychological and socioeconomic aspects of ageing in Poland. The subjects were randomly selected from 16 voivodships of Poland using three-stage, proportional sampling process stratified by gender and age group (spanning five years each: 55–59, 65–69, 70–74, etc), as described precisely elsewhere (27). The full PolSenior database included data gathered from 5’695 participants. Subjects were interviewed using a structured questionnaire; face to face interviews were conducted by pre-trained nurses.

The study fully complied with all applicable institutional and governmental regulations concerning the ethical use of human volunteers and with the terms of the Helsinki Declaration. The institutional review board approved the study protocol, and all the recruited subjects gave their written informed consent.

Data available for this analysis were limited to respondents over 65 years (N = 4’979). In a second step, we excluded from the analysis respondents for whom we have had no data for variables used in definition of healthy ageing. Finally, the sample considered in the presented analysis included 4’653 respondents (2’409 men and 2’244 women) whose mean age was 79.4 years (SD 8.72).

### Healthy ageing definitions

We have constructed three definitions of Healthy Ageing mainly based on Rowe-Khan model of successful ageing (5, 28). High cognitive and mental functioning was addressed by the Mini Mental State Examination Scale (MMSE; results of 24 points or higher indicate no cognitive impairment) and Geriatric Depression Scale (GDS, result of 6 or less points indicate no depressiveness (29)). The second component of Rowe-Khan concept — avoiding of disability was measured by Instrumental Activities of Daily Living scale (IADL; with maximum score of 24 points indicating being functionally active). The third one — social engagement was measured by the index of being socially active — the respondent was classified as socially active if he/she declared everyday going out or meeting friends/ neighbours at least once a week or participation in religious services at least once a week). The fourth component of healthy ageing — presence of diseases is the one which makes the three definitions different (Tab. 2). For the first definition we have not included any measure of the presence of disease, for the second one — only major chronic diseases were considered (i.e. stroke, cancer and Parkinson disease/epilepsy) and for the third definition presence of chronic diseases (classified as none or one disease and ≥1 diseases) was the indicator used. We have collected self-reported data about 14 chronic diseases: hypertension, arrhythmia, heart failure, coronary heart disease, stroke, Parkinson disease/epilepsy, cancer, chronic obstructive pulmonary disease, diabetes mellitus, chronic kidney disease, cataract, osteoporosis, diagnosed ever in respondent’s life and treatment for depression (ever in life) as well as stomach/duodenal ulcers and anaemia. The detailed description of our 3 definitions of HA is showed in Table 1.

**Table 1:** Different definitions of healthy ageing used in the present study

	**Definition 1**	**Definition 2**	**Definition 3**
Major chronic diseases*	NA	NO	NA
Chronic diseases			
(NO if 1 or no diseases**)	NA	NA	NO
Cognitive impairment			
(NO if MMSE ≥ 24/30)	NO	NO	NO
Depression (NO if GDS ≤ 6)	NO	NO	NO
Functionally active			
YES if IADL score = 24	YES	YES	YES
Socially active			
YES, if activity score = 1***	YES	YES	YES

NA — not applicable — the criterion was not taken into account for the specific definition; * Cancer, stroke, Parkinson disease/epilepsy; **Self-report of 14 chronic diseases: hypertension, arrhythmia, heart failure, coronary heart disease, stroke, Parkinson disease/epilepsy, cancer, chronic obstructive pulmonary disease, diabetes mellitus, chronic kidney disease, cataract, osteoporosis, diagnosed ever in respondent’s life and treatment for depression (ever in life) as well as acute diseases (stomach/duodenal ulcers and anaemia); *** Social activity score =1 if the respondent goes out everyday or meets friends/neighbours at least once a week or participates in religious services at least once a week

**Table 2:** Characteristics of the study participants

	**Total**	**Women**	**Men**	
	**N=4’653**	**N=2’244**	**N=2’409**	
	**n (%)**	**n (%)**	**n (%)**	**p**
Age (years)				
65–69	728 (15.7)	373 (16.6)	355 (14.7)	0.133
70–74	863 (18.60)	410 (18.3)	453 (18.8)	
75–79	779 (16.7)	372 (16.6)	407 (16.9)	
80–84	742 (16.0)	346 (15.4)	396 (16.4)	
85–89	814 (17.5)	371 (16.5)	443 (18.4)	
90+	727 (15.6)	372 (16.6)	355 (14.7)	
Education*				
Secondary School or higher	1’320 (28.4)	565 (25.3)	755 (31.4)	<0.001
Marital status*				
Married	2’273 (49.1)	599 (26.8)	1’674 (69.8)	<0.001
Place of residence				
Village	1’879 (40.4)	939 (41.8)	940 (39.0)	0.051
Employed*				
Yes	112 (2.4)	34 (1.5)	78 (3.2)	<0.001
Income (in PLZ)				
<=1’000	1’662 (35.7)	1’100 (49.0)	562 (23.3)	<0.001
1’001–2’000	2’029 (43.6)	834 (37.2)	1’195 (49.6)	
>2’000	393 (8.4)	66 (2.9)	327 (13.6)	
No response	569 (12.2)	244 (10.9)	325 (13.5)	
Shortage of money*				
Yes	977 (21.1)	569 (25.4)	408 (17.0)	<0.001
Physical exercising				
Yes	1’506 (32.4)	554 (24.7)	952 (39.5)	<0.001
Smoking				
Current smoker	419 (9.0)	95 (4.2)	324 (13.5)	<0.001
Self-rated health*				
Fair/good	1’579 (40.3)	685 (36.8)	894 (43.5)	<0.001
Falls during last year*				
Yes	1’059 (23.0)	590 (26.6)	469 (19.7)	<0.001
Need for help/care*				
Yes	1’898 (41.0)	1’035 (46.4)	863 (36.0)	<0.001

*missing values for: education — 13; marital status — 19; employment — 7; shortage of money — 15; self-rated health — 732; falls — 50; need of help — 26; PLZ — Polish Zlotys (1’000 PLZ is equivalent of about 300 USD)

In the WHO Report on healthy ageing much more indicators related to the healthy ageing concept have been proposed. WHO concept of healthy ageing is based on “functional ability, intrinsic capacity, subjective well-being, health characteristics, genetic inheritance, multimorbidity and the need for services and care” (7). In addition, in this model the environmental factors/influences should be also taken into account. To establish relation between different indicators of healthy ageing as proposed by WHO and our definitions we have examined the following variables available in our study:

- Education level — classified as having at least secondary education completed or not.
- Marital status — considered in two categories: married or not-married covering all other possibilities.
- Place of respondent’s residence — living in village vs. city/ town.
- The income — based on single question in the questionnaire, classified as a) <=1’000 PLZ (Polish Zlotys); b) 1’001–2’000 PLZ and c) > 2’000 PLZ. The cut points were defined in relation to the average retirement pay and pension in 2010 that was 1’642.92 PLZ (gross) and that resulted in about 1000 PLZ of disposable income [30]. The amount of 1’000 PLZ was an equivalent of about 300 USD. Because of a lot missing answers were found in this question we have decided to add additional category — no response.
- Shortage of money — based on respondent’s self-report of having enough money to cover basic costs of daily living (as food, medicines, clothes, etc.) — based on this question the variable “shortage of money” with answers YES or NO was constructed.
- Physical exercising — based on respondent’s declaration of performing at least once a week any of the following activities: exercising/aerobic or walking for at least an hour or swimming or biking.
- Smoking — assessed based on a single question “Do you smoke cigarettes regularly?” with YES/ NO answers possible
- Self-rated health (SRH) — originally measured at numerical scale ranging from 0 denoting the worst health status, to 10 denoting the best health status one could imagine. For the purpose of this analysis it was classified as good SRH if the scores were 7 or higher. These data were not available for respondents with cognitive impairment.
- History of falls during the previous year — based on a single question in questionnaire.
- Help needed form family or other caregivers as well as institutions — based on respondent’s subjective opinion (single question).

### Statistical Methods

Based on the data from the PolSenior project the appropriate weights to reflect the true distribution of gender, age and regions in the sampling procedure were constructed to assess the nationwide prevalence of health conditions in the Polish population of people aged 65 years and older (27). We have used these weights to assess the prevalence of healthy ageing according to different definitions used as well as conditions which constitutes these definitions. To compare our 3 definitions of HA with a set indicators proposed by WHO [7] first the tetrachoric correlations were used to account for dichotomous variables. Later, multivariate logistic regression models were used to establish the impact of these indicators on HA.

The STATA version 13 software was used to analyse data. The level of significance was set at α=0.05 for all analyses.

## Results

### Sample characteristics

**Figure 1:**
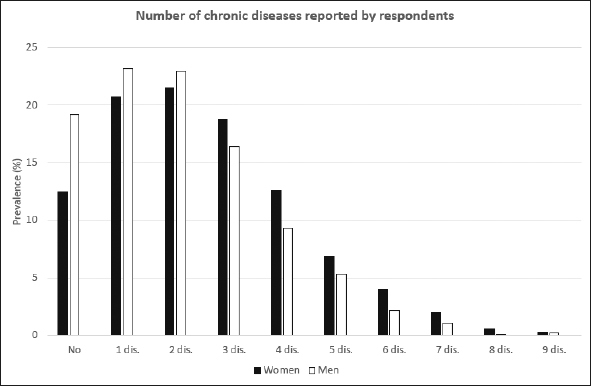
Number of chronic conditions reported by older respondents from PolSenior project — crude estimates

In our study the number of males was slightly higher than females (51.8% vs 48.2%), all age groups were represented approximately equally. Almost half of the respondents were married (49.1%). Over 28% of respondents had at least secondary education, most of them lived in town (59.6%), 32.4% of respondents declared to be physically active and only 9% were current smokers. Slightly over 40% of respondents assessed their health status as good. From the other side, 41% of studied population declared that they need a help from other people. Detailed description is given in Table 2. Distribution of number of chronic diseases (out of 14 studied diseases) is presented in Figure 1.

### Prevalence of Healthy ageing

The three definitions of healthy ageing were analysed — all three included good physical functioning, good cognitive functioning and no symptoms of depression as well as social engagement. The differences between those definitions were based on the inclusion of chronic conditions present. The first definition of HA (Def. I) does not include any measure of presence of chronic diseases, the second one (Def. II) assumes no healthy ageing if any of major chronic conditions (i.e. cancer, stroke, Parkinson disease/epilepsy) was present and the third definition (Def. III) allows for presence of no more than one chronic disease to be classifies as healthy ageing person (Tab. 1).

**Figure 2:**
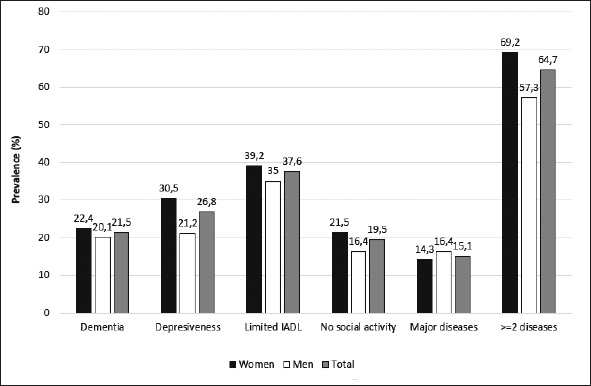
Estimates of population-based prevalence of conditions used for the definition of healthy ageing among Polish older citizens — weighted prevalence based on PolSenior data

As expected, the prevalence of healthy ageing varied by definition. In the table 3 the detailed information about prevalence of HA has been presented according to gender and age groups. Only in the youngest group and for the first two definitions of healthy ageing its prevalences have been found to be slightly higher among women then among men. In other groups the healthy ageing was found to be less prevalent among women. In addition, we have assessed the prevalence of healthy ageing in Polish population of people at age 65 years and older using the population weights established in the PolSenior project. The estimated weighted prevalence of healthy ageing was 42.8% for the first definition, 38.3% for the second and 17.6% for the third one. We have assessed that 64.7% of Polish elderly suffer from 2 or more chronic diseases (Figure 1), more often among women (69.2%) than men (57.3%). Out of the four conditions that were thought to limit HA in all our definitions limitations in instrumental activities of daily living was found to be most prevalent — it was estimated that 37.6% of the Polish older population are affected by this condition. Cognitive impairment was assessed to be present in 21.5% and depressiveness in 26.8% of Polish older citizens. Almost 20% of respondents seems to present no or very limited social activity (Figure 2).

**Table 3:** Prevalence of healthy ageing stratified by gender and age. Data are presented as prevalence together with 95% confidence intervals

**Gender**	**Age group**	**Def. I**	**Def. II**	**Def. III**
Females				
	65–69	57.9 (52.7–62.9)	52.8 (47.6–57.9)	25.1 (20.8–29.8)
	70–74	49.8 (44.8–54.7)	44.9 (40.1–50.0)	17.1 (13.6–21.1)
	75–79	30.6 (26.0–35.5)	27.0 (22.6–31.7)	9.7 (6.9–13.1)
	80–84	18.1 (14.3–22.5)	16.2 (12.5–20.4)	6.1 (3.9–9.1)
	85–89	6.4 (4.2–9.4)	5.9 (3.8–8.8)	2.1 (0.9–4.0)
	90+	2.3 (1.1–4.4)	2.1 (0.9–4.0)	0.8 (0.2–2.2)
Males				
	65–69	53.4 (48.0–58.6)	48.0 (42.8–53.4)	27.7 (23.1–32.6)
	70–74	50.3 (45.7–55.0)	45.5 (40.9–50.2)	22.4 (18.7–26.5)
	75–79	41.9 (37.1–46.8)	36.8 (32.2–41.7)	20.6 (16.8–24.8)
	80–84	30.1 (25.7–34.8)	25.2 (21.0–29.7)	10.4 (7.6–13.8)
	85–89	19.7 (16.1–23.6)	18.2 (14.7–22.0)	9.6 (7.1–12.7)
	90+	7.5 (5.1–10.7)	7.3 (4.9–10.4)	3.5 (1.9–5.9)

### Indicators of healthy ageing

The next step in our analysis was the examination of pairwise correlation among different predictors of HA and among those predictors and HA. We found only a few liaisons. The self-reported need for help/care from other people was strongly negatively correlated with physical activity (rho = −0.567) and good self-rated health (rho = −0.352) as well as being married (rho = −0.378); it was positively correlated with history of falls during the previous year (rho = 0.459). Moreover education level was strongly negatively correlated with living in a village (rho = −0.579). HA for all definitions was strongly correlated with self-assessed need for help/care (all rho < −0.7). The second strongest correlation was found for relation between HA and physical activity (rho = 0.553; 0.529 and 0.463 for the def. I, def. II and def. III, respectively) and the third one between HA and education (rho = 0.456; 0.405 and 0.279 for the def. I, def. II and def. III, respectively) — for both indicators the relation with the definition III of HA was lower than observed for the first two definitions. The opposite was observed for correlation between HA and good self-rated health. The stronger correlation was found between good SHR and third definition of HA (rho = 0.438) than SHR and other two definitions (rho = 0.353 for def. I and rho = 0.369 for def. II).

For the first two definitions of HA we identified set of similar independent predictors: higher odds of HA was found for married respondents, higher income, physical activity, higher SRH and lower odds of HA was related to older age, smoking, experience of falls during last year and need for help/care. In addition, living in village and smoking were related to lower odds of HA, but only for the first definition. The inclusion of absence of major diseases (cancer, stroke or Parkinson disease/epilepsy) in definition of HA (Def. II) implied the increase of strength of association (as compared with Def. I) for marital status (OR=1.48 vs OR=1.38 for Def. II and Def. I respectively), income (OR = 1.76 vs OR = 1.61 for the > 2’000 PLZ group) and self-rated health (OR = 1.99 vs OR = 1.87) and decrease of ORs for education (OR = 1.93 vs OR = 2.42) and physical exercising (OR = 1.93 vs OR = 2.10) as compared to the Def. I. In addition, the odds of HA defined by Def. I was lower for current smokers (OR = 0.76; 95%CI: 0.59–0.99; Tab. 4). For the third definition of HA (in which presence of 2 or more diseases means no HA) we have showed that age, education marital status physical activity, SRH and need for care/help but neither falls during last year nor income were significant predictors of HA. However, additional predictor of healthy ageing was self-reported shortage of money in the household.

The separate analysis for men and women showed similar predictors of healthy ageing to be important. The differences were related to the strength of associations observed. Among men aged 85 and older the impact of age on HA was weaker and for women in the same age groups stronger than those observed for the whole group, and this observation was independent of the definition used. The stronger relationships among men than among women were also found for education and opposite weaker associations were found for marital status, physical exercising and self-rated health. Differences between men and women were also found for impact of income on healthy ageing, especially for the first two definitions — for men higher OR were found for the income category 1’001-2’000 PLZ and smaller for the income >2’000 PLZ then for women. Detailed results for the men and women are presented in the Annex.

A self-assessed need for help/care from other people or institution was found to be the strongest independent predictor of lack of healthy ageing. It reduced the odds of healthy ageing more than six-folds and this relation was equally strong for all definitions as well as for men and women (see Appendix).

## Discussion

We examined three definitions of healthy ageing — based on Rowe and Kahn concept of successful ageing (5) — with its physical component — disease and disability, good physical and cognitive functioning as well as social and productivity engagement. All these elements were included in the definitions proposed — we included measure of good physical functioning measured by Instrumental Activities of Daily Living scale (IADL), cognitive functioning measured by Mini Mental State Examination (MMSE) and the symptoms of depression (based on Geriatric Depression Scale — GDS) as well as indicator of social engagement measured by participation in religious services or meeting other people by going out — those activities were previously found to be important predictors of both health status (mortality, morbidity, cognitive functioning and physical functioning) and self-rated health (17–19).

We found that presence of healthy ageing strongly depends on the definition but across definitions, the odds of healthy ageing were lower for older age groups, less educated and not married ones. In addition, the lower ORs were also lower for respondents with lower income (for def. I and II) or those who declared “shortage of money” (for def. III only). The Moreover, we observed that the strongest factor negatively influencing healthy ageing was respondent’s perception of need for help or care from other people.

Many researchers as well as laypersons argued that the healthy or successful ageing does not mean “without disease” (9, 20). At older age over 80% of people seems to have at least one chronic condition (21, 22), some of them last for decades and people “used to” live with them. Many older people consider themselves to have aged successfully, whereas classifications based on health status indicators do not. The element that makes our definitions different from each other is presence of chronic diseases — the first one does not included information about diseases, in the second definition only major diseases were treated as limitation to healthy ageing, and the third one assumes that presence of two or more diseases means that person should not be described as healthy older person. The prevalence of healthy ageing in the Polish population aged 65 years and older was estimated to be from 17.6% to 42.8% based on definition used. In other studies the prevalence of healthy or successful ageing was found very heterogeneous — it depends, first of all, on definition used and on the population characteristics, including some cultural perspective. Based on the SHARE data the prevalence of healthy ageing in Polish older population was estimates to be from less than 2% to more than 40% (Polish and Hungarian data together) (16, 17)

Our findings are consistent with a broad literature showing higher prevalence of health problems and functional limitations among those of advance age, and characterized with lower socioeconomic status (10, 13–15, 23). This suggests that our definitions capture known health disadvantages in older populations. In our study there was not consistent impact of gender on odds of healthy ageing — that relation depended on definition of HA that was used. Only in the model in which the definition of HA covers absence of major chronic diseases (def. II) men had lower odds of being HA than women did. In the other studies the relation between gender and HA is not clear, it depends on definition. Some studies showed that women had lower odds of experiencing healthy ageing (15, 24), other showed opposite relation (13) and some studies did not confirmed any association between gender and healthy ageing (10).

We have also confirmed the strong impact of physical exercises on chance to experience healthy ageing. People who perform physical exercises have higher functional activity, better health status, and more possibilities to have social relations, to go outside or to meet other people, so higher chance to be healthy person. This finding is consistent with other studies showing similar strength of association (6).

Using each of healthy ageing definitions allows us to identify group of people who rated their health as good providing evidence that all of these definitions can be valid. Stronger relation between self-rated health and healthy ageing exists when the existence of diseases was included in the definition. However, one should remember that healthy ageing is not equivalent of self-rated health. Self-rated health is a subjective measure and it depends on respondent’s perception of own’s experiences from the past both related to health status of himself or people in the closest environment and also his personality and expectations (25). From the other side “healthy ageing” should be based on some objective measures to allow its comparability between different population groups.

In our study we have observed that the strongest factor influencing healthy ageing was self-assessment as a person who needs help or care from other people. This is in line with the layperson perspective — that the most important from their point of view is independence and possibility to take care about themselves (26).

Some limitation of our study should be mentioned. First, data used in this study were self-reported, and thus are subject to measurement errors, especially those related to ability to recall some information as well as recognition ability. We used also some data collected from proxy respondents (caregivers), however there were small amount of interviews (less than 7%) for which care giver was the main source of information about the respondents. The participation rate in PolSenior Project (for the whole study) was only 42% and this may lead to high risk of selection bias. We should also mention that the respondents who decided to participate in the study may have relatively good functional status and were generally healthier than the general population in this age group in Poland, thus estimated prevalence of Healthy aging based on these data can be overestimated and should be treated with caution.

However, our data were collected in a nation-wide cross-sectional study with sampling procedure allowing for assessment of all gender and age group. Oversampling of the older groups and men (as compared to the proportion in the Polish population at age 65 years and older) allowed to study health outcomes in all groups. Subsequently, the prevalence data could be generalized for the Polish population using weighting procedure to receive representative population.

Based on results from our study, healthy ageing seems to be best described as a status of being socially active with no functional limitations, no more than 2 diseases and neither cognitive impairment nor depressiveness. Additionally, we have observed an impact of health predictors like: age, marital status, education and income on the odds of healthy ageing and it confirms that proposed definition fits to tendencies observed in population. Secondly, the definition showed to be related to the indicators of physical functioning (falls during last year) and lifestyle (physical exercising). Finally, we have found good concordance with self-rated health measure as well as the self-assessed need for help.

## Electronic supplementary material

Supplementary material, approximately 16.9 KB.

## Notes

*Ethical Standards:* The PolSenior study was approved by the Bioethics Commission of the Medical University of Silesia in Katowice (ethical approval no. KNW-6501-38/I//08)
