# Supplementary material for: Influence of Sociodemographic, Behavioral and Other Health-Related Factors on Healthy Ageing Based on Three Operative Definitions
Source: J Nutr Health Aging. 2019 Aug 30;23(9):862–9. doi: 10.1007/s12603-019-1243-5 (PMC6800399; doi:10.1007/s12603-019-1243-5)
Supplement: Supplementary file 1 — Supplementary material, approximately 16.9 KB. [file mmc1.docx]

Table 4: Predictors of healthy ageing (defined according three definitions) in the PolSenior female population aged 65+ years

Category **Def. I Def. II Def. III**

**Age (years)**

65-69 1.00 (ref.) 1.00 (ref.) 1.00 (ref.)

70-74 0.85 (0.61-1.19) 0.86 (0.62-1.20) 0.75 (0.51-1.11)

75-79 0.56 (0.39-0.81) 0.57 (0.39-0.82) 0.58 (0.36-0.91)

80-84 0.50 (0.33-0.77) 0.53 (0.34-0.82) 0.59 (0.34-1.05)

85-89 0.18 (0.10-0.31) 0.20 (0.11-0.36) 0.26 (0.12-0.59)

90+ 0.10 (0.05-0.22) 0.12 (0.05-0.26) 0.16 (0.05-0.53)

**Education**

Secondary School or higher 1.97 (1.46-2.46) 1.73 (1.28-2.33) 1.18 (0.81-1.72)

**Marital status**

Married 1.43 (1.09-1.88) 1.42 (1.09-1.86) 1.31 (0.94-1.82)

**Place of residence**

Village 0.79 (0.60-1.04) 0.84 (0.64-1.11) 0.98 (0.69-1.39)

**Employed**

Yes 1.27 (0.47-3.47) 1.33 (0.51-3.44) 1.06 (0.41-2.76)

**Income (in PLZ)**

<=1000 1.00 (ref.) 1.00 (ref.) 1.00 (ref.)

1001-2000 1.13 (0.83-1.53) 1.29 (0.95-1.74) 1.21 (0.82-1.78)

>2000 3.03 (1.39-6.61) 2.56 (1.20-5.46) 1.30 (0.56-3.03)

No response 0.89 (0.57-1.39) 1.09 (0.70-1.70) 1.02 (0.57-1.81)

**Shortage of money**

Yes 0.78 (0.60-1.04) 0.84 (0.63-1.12) 0.59 (0.40-0.88)

**Physical exercising**

Yes 2.65 (2.05-3.43) 2.36 (1.83-3.04) 2.05 (1.50-2.81)

**Smoking**

Current smoker 0.74 (0.44-1.27) 0.94 (0.56-1.59) 1.34 (0.74-2.42)

**Self-rated health**

Fair/good 1.99 (1.55-2.55) 2.02 (1.57-2.58) 2.90 (2.11-3.97)

**Falls during last year**

Yes 0.65 (0.47-0.91) 0.61 (0.43-0.85) 0.93 (0.60-1.46)

**Need for help/care**

Yes 0.16 (0.11-0.22) 0.16 (0.11-0.23) 0.14 (0.07-0.27)

PLZ – Polish Zlotys (1’000 PLZ is equivalent of about 300 USD)

Table 5: Predictors of healthy ageing (defined according three definitions) in the PolSenior male population aged 65+ years

Category **Def. I Def. II Def. III**

**Age (years)**

65-69 1.00 (ref.) 1.00 (ref.) 1.00 (ref.)

70-74 0.95 (0.67-1.32) 0.98 (0.71-1.35) 0.83 (0.58-1.17)

75-79 0.68 (0.48-0.91) 0.69 (0.49-0.97) 0.79 (0.54-1.13)

80-84 0.51 (0.35-0.73) 0.50 (0.34-0.72) 0.44 (0.28-0.69)

85-89 0.47 (0.32-0.69) 0.57 (0.39-0.85) 0.74 (0.47-1.16)

90+ 0.22 (0.13-0.38) 0.32 (0.19-0.54) 0.42 (0.22-0.83)

**Education**

Secondary School or higher 2.74 (2.13-3.52) 2.06 (1.60-2.63) 1.42 (1.14-1.77)

**Marital status**

Married 1.29 (1.00-1.67) 1.50 (1.15-1.95) 1.58 (1.19-2.10)

**Place of residence**

Village 0.79 (0.62-1.02) 0.86 (0.67-1.10) 0.99 (0.74-1.33)

**Employed**

Yes 0.92 (0.53-1.62) 0.86 (0.50-1.48) 0.99 (0.57-1.73)

**Income (in PLZ)**

<=1000 1.00 (ref.) 1.00 (ref.) 1.00 (ref.)

1001-2000 1.42 (1.05-1.90) 1.44 (1.07-1.94) 1.21 (0.85-1.72)

>2000 1.50 (0.99-2.26) 1.64 (1.09-2.46) 1.18 (0.74-1.89)

No response 1.15 (0.77-1.70) 0.94 (0.63-1.41) 0.79 (0.48-1.28)

**Shortage of money**

Yes 0.84 (0.62-1.13) 0.85 (0.63-1.14) 0.61 (0.41-0.89)

**Physical exercising**

Yes 1.78 (1.43-2.21) 1.67 (1.34-2.07) 1.70 (1.32-2.20)

**Smoking**

Current smoker 0.81 (0.59-1.10) 0.87 (0.64-1.19) 1.32 (0.94-1.85)

**Self-rated health**

Fair/good 1.79 (1.44-2.22) 1.98 (1.60-2.45) 2.63 (2.04-3.39)

**Falls during last year**

Yes 0.66 (0.48-0.92) 0.65 (0.46-0.91) 0.77 (0.50-1.19)

**Need for help/care**

Yes 0.14 (0.10-0.20) 0.13 (0.09-0.19) 0.13 (0.07-0.23)

PLZ – Polish Zlotys (1’000 PLZ is equivalent of about 300 USD)
